# Real-World Genomic Landscape of Korean Gastric Cancer: Integrating Biomarker Associations and Clinical Outcomes in Metastatic Gastric Cancer

**DOI:** 10.1200/PO-25-01090

**Published:** 2026-02-19

**Authors:** Seong-Keun Yoo, Soomin Ahn, Jinha Hwang, Jongwu Kim, Yunjin Go, Minsuk Kwon, Sung Hee Lim, Seung Tae Kim, Kyoung-Mee Kim, Jeeyun Lee

**Affiliations:** ^1^ Samsung Precision Genome Medicine Institute, Samsung Medical Center, Seoul, Republic of Korea; ^2^ Division of Hematology-Oncology, Department of Medicine, Samsung Medical Center, Sungkyunkwan University School of Medicine, Seoul, Republic of Korea; ^3^ Department of Pathology and Translational Genomics, Samsung Medical Center, Sungkyunkwan University School of Medicine, Seoul, Republic of Korea

## Abstract

**PURPOSE:**

This study aimed to characterize the genomic landscape of Korean gastric cancer and evaluate associations among oncogenic alterations, established biomarkers, demographics, and treatment outcomes.

**METHODS:**

A total of 1,283 patients with gastric cancer who underwent tumor-only targeted sequencing as part of practice and received palliative treatment between January 2017 and August 2025 at the Samsung Medical Center were included.

**RESULTS:**

Among 1,283 patients (median [IQR] age, 61 [52-68] years; 827 males [64.46%]), *TP53* (51.91%), *ARID1A* (19.02%), *ERBB2* (12%), *KRAS* (10.29%), and *PIK3CA* (9.12%) were the most frequently altered genes. Epstein-Barr virus–positive tumors exhibited enrichment of *BCOR*, *PIK3CA*, and *ARID1A* alterations and reduced *TP53* mutations (false discovery rate [FDR] adjusted *P* < .01). Human epidermal growth factor receptor 2–positive tumors were characterized by coamplification of *ERBB2*, *CCNE1*, and *MYC* (FDR adjusted *P* < .001), whereas PD-L1 positivity was associated with *KRAS* and *CDKN2A* alterations (FDR-adjusted *P* < .05). Among patients treated with first-line nivolumab plus chemotherapy (n = 269), those with high tumor mutational burden (TMB; ≥10 mutations per megabase) had improved overall survival (*v* the low TMB subgroup; hazard ratio [HR], 0.48 [95% CI, 0.25 to 0.93]; *P* = .03), particularly when combined with PD-L1 positivity (*v* all other biomarker-defined subgroups; HR, 0.33 [95% CI, 0.14 to 0.76]; *P* = .006). Moreover, as TMB levels increased, patients derived greater survival benefit from nivolumab plus chemotherapy versus chemotherapy alone, even among those with microsatellite-stable tumors. Across treatment regimens, *FGFR2* and *MET* alterations were linked to poorer outcomes, whereas *PIK3CA* mutations were observed in patients with longer overall survival after first-line chemotherapy.

**CONCLUSION:**

Our findings provide a comprehensive genomic landscape of Korean gastric cancer and underscore the clinical relevance of integrating genomic and established biomarkers to advance precision oncology.

## INTRODUCTION

Gastric cancer is a leading cause of cancer-related mortality worldwide, particularly in East Asia, where incidence and mortality continue to remain high.^1^ Despite advances in surgery and systemic therapy, outcomes for advanced disease remain unsatisfactory, with median overall survival rarely exceeding 1 year under conventional chemotherapy.^2,3^ These limitations have prompted a shift toward molecularly defined subtypes and biomarker-driven treatment strategies to improve therapeutic precision.^4^

**CONTEXT**

**Key Objective**
What is the genomic landscape of Korean gastric cancer, and how it is associated with established biomarkers, demographics, and treatment outcomes in real-world data?
**Knowledge Generated**
In 1,283 patients, biomarker-defined subgroups exhibited distinct genomic profiles. *CDH1* alterations were enriched in young female patients. Costratification of PD-L1 and tumor mutational burden (TMB) identified a subgroup of patients with prolonged overall survival after first-line nivolumab plus chemotherapy. The TMB-high subgroup benefited more from first-line nivolumab plus chemotherapy over chemotherapy alone, even among patients with microsatellite-stable disease. Associations between specific genes and overall survival were observed across different treatment settings.
**Relevance**
Integrating genomic and established biomarkers can advance precision oncology in gastric cancer.

Traditional histologic classifications such as the Lauren or WHO schemes inadequately reflect the biological heterogeneity of gastric cancer.^5^ Genomic profiling efforts, most notably by The Cancer Genome Atlas (TCGA) and the Asian Cancer Research Group (ACRG), have revolutionized the molecular understanding of this disease.^6,7^ The TCGA proposed four molecular subtypes (Epstein-Barr virus [EBV]–positive, microsatellite instability [MSI]-high [MSI-H], genomically stable [GS], and chromosomal instability [CIN]), revealing recurrent actionable alterations such as *ERBB2*, *PIK3CA*, and *ARID1A*.^6^ By contrast, the ACRG, focusing on East Asian patients, identified MSI, microsatellite-stable (MSS)/epithelial-mesenchymal transition (EMT), MSS/TP53^+^, and MSS/TP53^–^ subtypes, linking distinct molecular profiles with prognostic differences, including the poor outcomes of MSS/EMT tumors.^7^ These frameworks underscore geographic and ancestry-related diversity in gastric cancer biology, yet translation into clinical practice remains limited because of cost, complexity, and under-representation of non-European ancestry populations.^8^

Over the past decade, several pivotal phase III trials have reshaped the systemic treatment landscape. The CheckMate 649 trial established nivolumab plus fluoropyrimidine-oxaliplatin chemotherapy as a first-line standard for advanced gastric, gastroesophageal junction, and esophageal adenocarcinoma, demonstrating significantly improved overall and progression-free survival compared with chemotherapy alone.^9^ Subsequent landmark trials, including ATTRACTION-4, KEYNOTE-590, and KEYNOTE-811, further confirmed the durable benefit of immune checkpoint inhibitor (ICI)–based combination therapy in these malignancies.^10-12^ Importantly, these trials have demonstrated that PD-L1 expression, MSI, and tumor mutational burden (TMB) serve as predictive biomarkers for identifying patients most likely to benefit from treatments.^10-13^ Collectively, these data illustrate the evolving biomarker-guided therapeutic paradigm.

Real-world data (RWD), derived from electronic health records, clinical data warehouses, and national cancer registries, have become indispensable complements to clinical trials.^14-16^ RWD captures heterogeneous patient populations often excluded from prospective studies and can validate trial findings under routine clinical conditions. Recent analyses demonstrated that RWD-driven molecular and clinical analyses can replicate pivotal trial results, generate new biomarker hypotheses, and inform regulatory decisions.^17-21^ The US Food and Drug Administration (FDA) and the European Medicines Agency now recognize RWD and real-world evidence as critical components for postapproval surveillance, label expansion, and health technology assessment.^22,23^ In oncology, RWD-driven studies have provided key insights into treatment sequencing, resistance mechanisms, and genomic evolution under therapy, dimensions often unobservable in tightly controlled trials.^24-26^

In this study, we leveraged single-center RWD at the Samsung Medical Center, integrating targeted sequencing results with detailed clinical and biomarker data. In Korea, the clinical sequencing tests had been implemented as part of practice since 2017 for patients with metastatic cancer.^27^ This real-world genomic data set, encompassing 1,283 patients with stage IV gastric cancer, provides one of the largest data sets to examine the molecular landscape in an East Asian population. Specifically, we sought to (1) delineate the mutational spectrum of Korean gastric cancer, (2) explore enrichment of oncogenic alterations across biomarker-defined subgroups, (3) characterize demographic associations, (4) evaluate the combined predictive value of PD-L1 and TMB in identifying patients with superior outcomes on first-line nivolumab plus chemotherapy, and (5) identify prognostic alterations across treatment contexts. Through integration of genomic, biomarker, and real-world clinical outcomes, this study provides an actionable molecular framework for precision oncology in gastric cancer.

## METHODS

This study was approved by the Institutional Review Board (IRB) of the Samsung Medical Center (IRB no. 2021-09-052). All patients who participated in this study provided written informed consent before enrollment and specimen collection. For comprehensive methods and materials, see the Data Supplement.

## RESULTS

### Study Flow and Treatment Distribution

**FIG 1.**
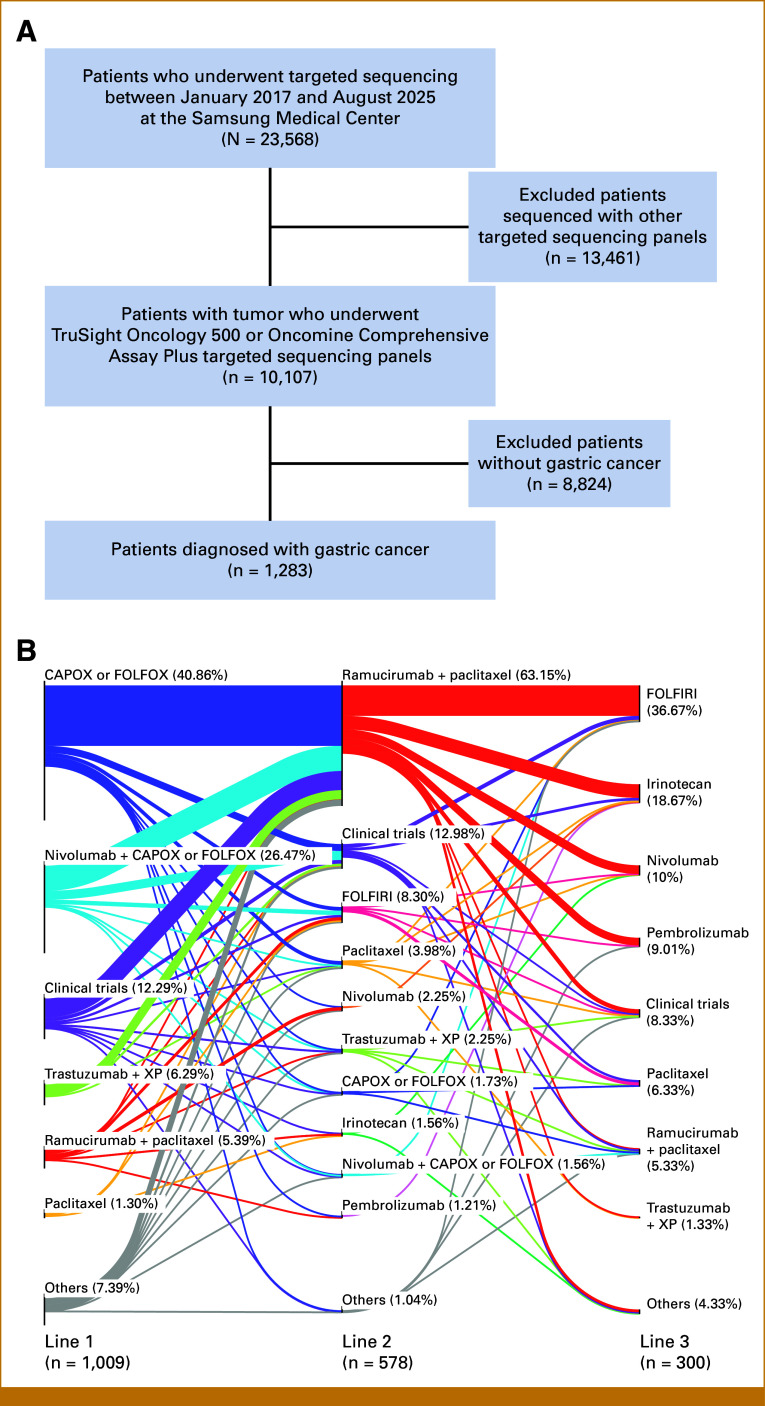
Study population. (A) CONSORT diagram describing inclusion and exclusion criteria of the study cohort. (B) Sankey diagram illustrating treatment patterns and sequential therapy transitions across first-, second-, and third-line treatments in the study cohort. Starting with an initial cohort of 1,283 patients, we applied a sequential filtering process to ensure data integrity for treatment course summarization. We excluded patients with missing line data, and those lacking first-line treatment records. In addition, patients with discontinuous treatment histories (eg, skipping from line 1 to line 3) or discordance between regimen codes and regimen names were removed. Consequently, the final Sankey plot visualizes the treatment trajectories of the remaining 1,009 patients. CAPOX, capecitabine and oxaliplatin; FOLFOX, infusional fluorouracil, leucovorin, and oxaliplatin.

A total of 23,568 patients underwent targeted sequencing between January 2017 and August 2025 at the Samsung Medical Center. After excluding 13,461 patients who received sequencing with other targeted panels (<500 genes) and 8,824 patients who did not have gastric cancer, we analyzed the mutational landscape of 1,283 stage IV gastric cancers using two targeted sequencing panels which included more than 500 genes (Fig 1A). The median age of the cohort was 61 years (IQR, 52-68), and 64.46% was male (n = 827). Among patients with available test results, the prevalence of biomarkers with therapeutic significance in gastric cancer^28,29^ was as follows: EBV, 4%; MSI-H, 4.13%; human epidermal growth factor receptor 2 (HER2), 9.04%; PD-L1, 75.62%; and CLDN18.2, 39.25% (Table 1).

**TABLE 1.** Patient Characteristics

Characteristic	Total Patients (n = 1,283)
Sex, No. (%)	
Female	456 (35.54)
Male	827 (64.46)
Age, years	
Median (IQR)	61 (52-68)
Overall survival, months	
Median (95% CI)	16.73 (15.12 to 18.61)
ECOG-PS, No. (%)	
0	208 (16.21)
1	1,032 (80.44)
2	37 (2.88)
3	6 (0.47)
HER2, No. (%)	
Positive	100 (9.04)^a^
Negative	1,006 (90.96)^a^
Not available	177
Microsatellite instability, No. (%)	
High	53 (4.13)
Stable	1,230 (95.87)
PD-L1, No. (%)	
Positive	729 (75.62)^a^
Negative	235 (24.38)^a^
Not available	319
EBV, No. (%)	
Positive	37 (4)^a^
Negative	888 (96)^a^
Not available	358
CLDN18.2, No. (%)	
Positive	73 (39.25)^a^
Negative	113 (60.75)^a^
Not available	1,097

Abbreviations: EBV, Epstein-Barr virus; ECOG-PS, Eastern Cooperative Oncology Group performance status; HER2, human epidermal growth factor receptor 2.

^a^ Percentages calculated among patients with available test results.

Patients received a median of two lines of therapy; treatment distribution across lines of therapy is summarized in Figure 1B. Among first-line regimens, palliative capecitabine and oxaliplatin (CAPOX) or infusional fluorouracil, leucovorin, and oxaliplatin (FOLFOX) (40.86%) was the most common, followed by nivolumab plus CAPOX or FOLFOX (26.47%), trastuzumab plus XP (6.29%), and ramucirumab plus paclitaxel (5.39%). In the second-line setting, ramucirumab plus paclitaxel (63.15%) predominated, with smaller proportions receiving CAPOX or FOLFOX (1.73%) and nivolumab-based combinations (1.56%). In the third-line setting, fluorouracil, leucovorin, and irinotecan (36.67%), irinotecan (18.67%), and nivolumab monotherapy (10%) were most frequent. Clinical trial enrollment rates were 12.29%, 12.98%, and 8.33% in the first-, second-, and third-line settings, respectively. Overall, 20.91% of patients participated in at least one clinical trial during their treatment course. The median overall survival of all patients with metastatic gastric cancer was 16.73 months (Table 1).

### Mutational Landscape of Korean Stage IV Gastric Cancer

**FIG 2.**
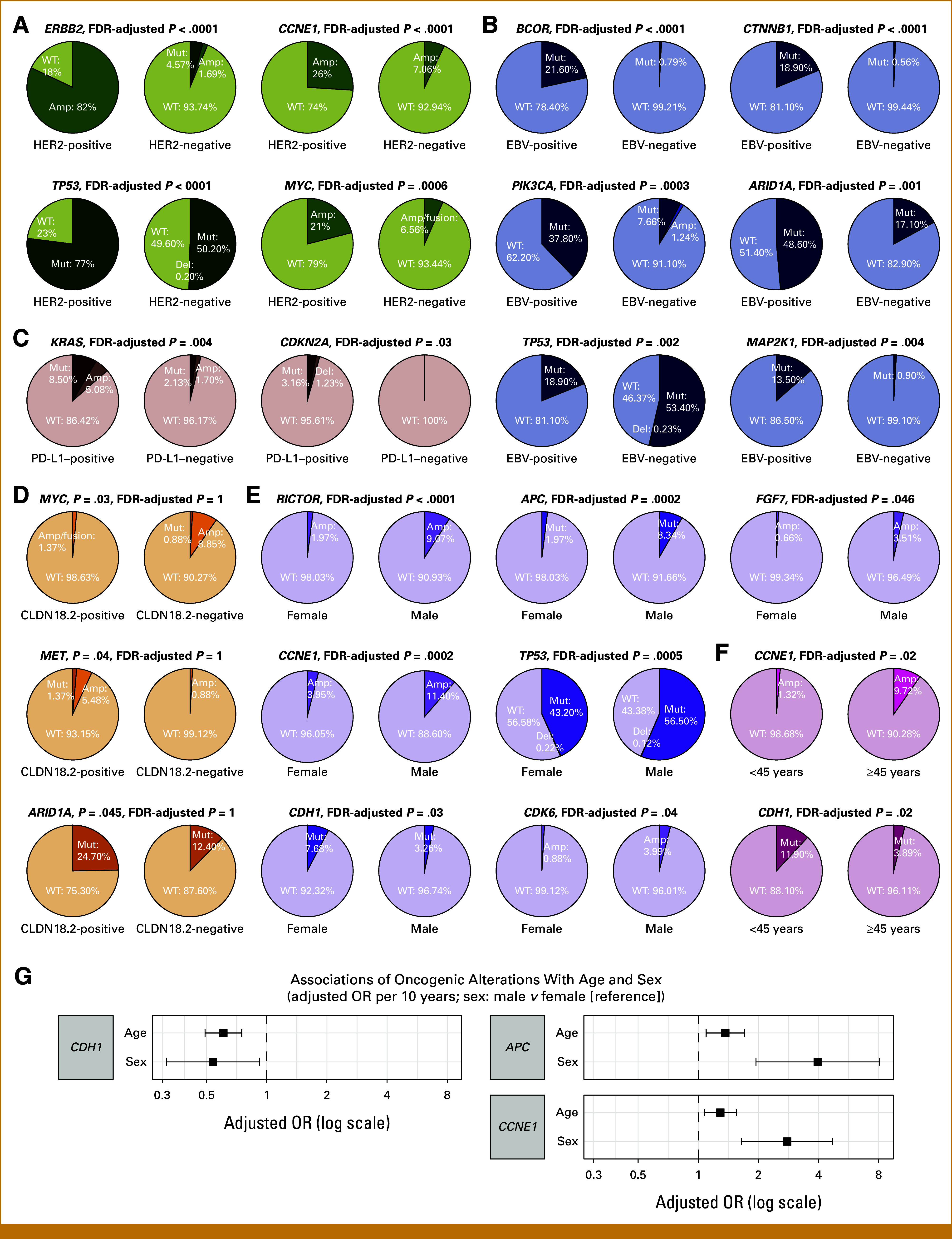
Oncogenic alterations associated with biomarker-defined and demographic subgroups. Pie charts displaying the proportion of altered (dark shade) versus wild-type (light shade) tumors for genes associated with biomarker-defined or demographic subgroups: (A) HER2-positive versus HER2-negative, (B) EBV-positive versus EBV-negative, (C) PD-L1–positive versus PD-L1–negative, (D) CLDN18.2-positive versus CLDN18.2-negative, (E) female versus male, and (F) young (<45 years) versus old (≥45 years). *P* values from two-sided Fisher's exact tests were adjusted using the Benjamini-Hochberg FDR procedure. Co-occurring SNV/indel and CNA were assigned to the CNA group. Genes associated with HER2, EBV, PD-L1, age, and sex met FDR-adjusted significance threshold (<.05), whereas those stratified by CLDN18.2 status met the raw *P* value significance criteria (<.05) because of limited subgroup size. (G) Adjusted ORs with 95% CIs from multivariable logistic regression models assessing gene-level associations with age and sex. Each panel represents a different gene with adjusted ORs plotted on a log scale. For age, adjusted ORs represent the change in gene alteration odds per 10-year increase. For sex, adjusted ORs compare males versus females (reference). Amp, amplification; CNA, copy number alteration; Del, deletion; EBV, Epstein-Barr virus; FDR, false discovery rate; HER2, human epidermal growth factor receptor 2; Mut, mutations including SNVs or small insertions and deletions (indels); OR, odds ratio; SNV, small nucleotide variant.

The oncoprint illustrates the distribution and frequency of recurrently altered genes across the cohort (Data Supplement, Fig S1). The most frequently mutated genes were *TP53* (51.91%), *ARID1A* (19.02%), *ERBB2* (12%), *KRAS* (10.29%), *PIK3CA* (9.12%), *CCNE1* (8.73%), *MYC* (8.26%), *RICTOR* (6.55%), *RHOA* (6.47%), and *APC* (6.08%). The incidence of *ERBB2* amplification, *FGFR2* amplification/fusion, and *MET* amplification/fusion was 8.26%, 5.14%, and 3.51%, respectively. Among 1,106 patients with available test results, the concordance rate of *ERBB2* amplification from sequencing and HER2 immunohistochemistry positivity was 82% (Fig 2A). *CCNE1* (26%), *TP53* (77%), and *MYC* (21%) alterations were associated with HER2-positive tumors (*v* 7.06%, 50.40%, and 6.56% in HER2-negative tumors; false discovery rate [FDR]–adjusted *P* < .0001, <.0001, and .0006, respectively). When comparing mutation frequencies based on EBV status (n = 925), *BCOR* (21.60%), *CTNNB1* (18.90%), *PIK3CA* (37.80%), *ARID1A* (48.60%), and *MAP2K1* (13.50%) were predominantly altered in EBV-positive tumors (*v* 0.79%, 0.56%, 8.90%; 17.10%, and 0.90% in EBV-negative tumors; FDR-adjusted *P* < .0001, <.0001, .0003, .001, and .004, respectively), whereas *TP53* (18.90%) alterations were less common in EBV-positive tumors (*v* 53.63% in EBV-negative tumors; FDR-adjusted *P* = .002; Fig 2B). These findings were consistent with TCGA analyses, which also reported enrichment of *BCOR*, *PIK3CA*, and *ARID1A* mutations, but a lack of *TP53* mutations, in EBV-positive tumors.^6^ Analysis of PD-L1 expression status (n = 964) revealed that *KRAS* (13.58%) and *CDKN2A* (4.39%) alterations were more frequent in PD-L1–positive tumors (*v* 3.83% and 0% in PD-L1–negative tumors, FDR-adjusted *P* = .004 and .03, respectively; Fig 2C). The Cochran-Mantel-Haenszel test confirmed a significant association between PD-L1 positivity and *KRAS* alterations, independent of MSI status (common odds ratio [OR], 3.68 [95% CI, 1.83 to 7.42]; *P* = .0002; Data Supplement, Fig S2). *MET* and *ARID1A* were more prevalent in CLDN18.2-positive tumors (6.85% and 24.70% *v* 0.88% and 12.40% in CLDN18.2-negative tumors; Fig 2D), whereas *MYC* was enriched in CLDN18.2-negative tumors (9.73% *v* 1.37% in CLDN18.2-positive tumors). However, none of these associations remained statistically significant after FDR correction. Because CLDN18.2 testing has been introduced in practice since 2024, test results were available for just 14.50% (n = 186) of patients in our cohort. As the number of tested cases increases, more accurate estimates of these associations are expected.

For demographic variables, we first compared alteration frequencies stratified by sex or age groups (<45 *v* ≥45 years). Several genes showed significant differences in mutation prevalence between males and females (*RICTOR*, *APC*, *CCNE1*, *TP53*, *CDH1*, *CDK6*, and *FGF7*; Fig 2E) or between younger and older patients (*CCNE1* and *CDH1*; Fig 2F). To further identify gene alterations simultaneously associated with both age and sex, we performed multivariable logistic regression with the two demographic variables as covariates (Fig 2G). Among the genes associated with age or sex, *CDH1* alterations were significantly associated with younger age (adjusted OR, 0.61 per 10-year increase [95% CI, 0.49 to 0.75]) and female sex (adjusted OR, 0.54 for male *v* female [reference] [95% CI, 0.31 to 0.92]). By contrast, *APC* alterations were significantly associated with both older age (adjusted OR, 1.37 [95% CI, 1.09 to 1.71]) and male sex (adjusted OR, 3.95 [95% CI, 1.94 to 8.04]). Similarly, *CCNE1* amplifications were more common in older male patients, with increased odds associated with age (adjusted OR, 1.29 [95% CI, 1.07 to 1.55]) and male sex (adjusted OR, 2.79 [95% CI, 1.65 to 4.70]).

### Biomarker Analysis for First-Line Nivolumab Plus Chemotherapy

Nivolumab plus chemotherapy has recently been approved as a first-line standard regimen for advanced gastroesophageal adenocarcinoma in many countries.^30^ Consistent with this, a substantial proportion of patients (n = 269) in our cohort also received nivolumab plus chemotherapy (CAPOX or FOLFOX) as first-line treatment. Therefore, biomarker analyses were first conducted within this chemoimmunotherapy group to determine factors associated with improved therapeutic benefit.

**FIG 3.**
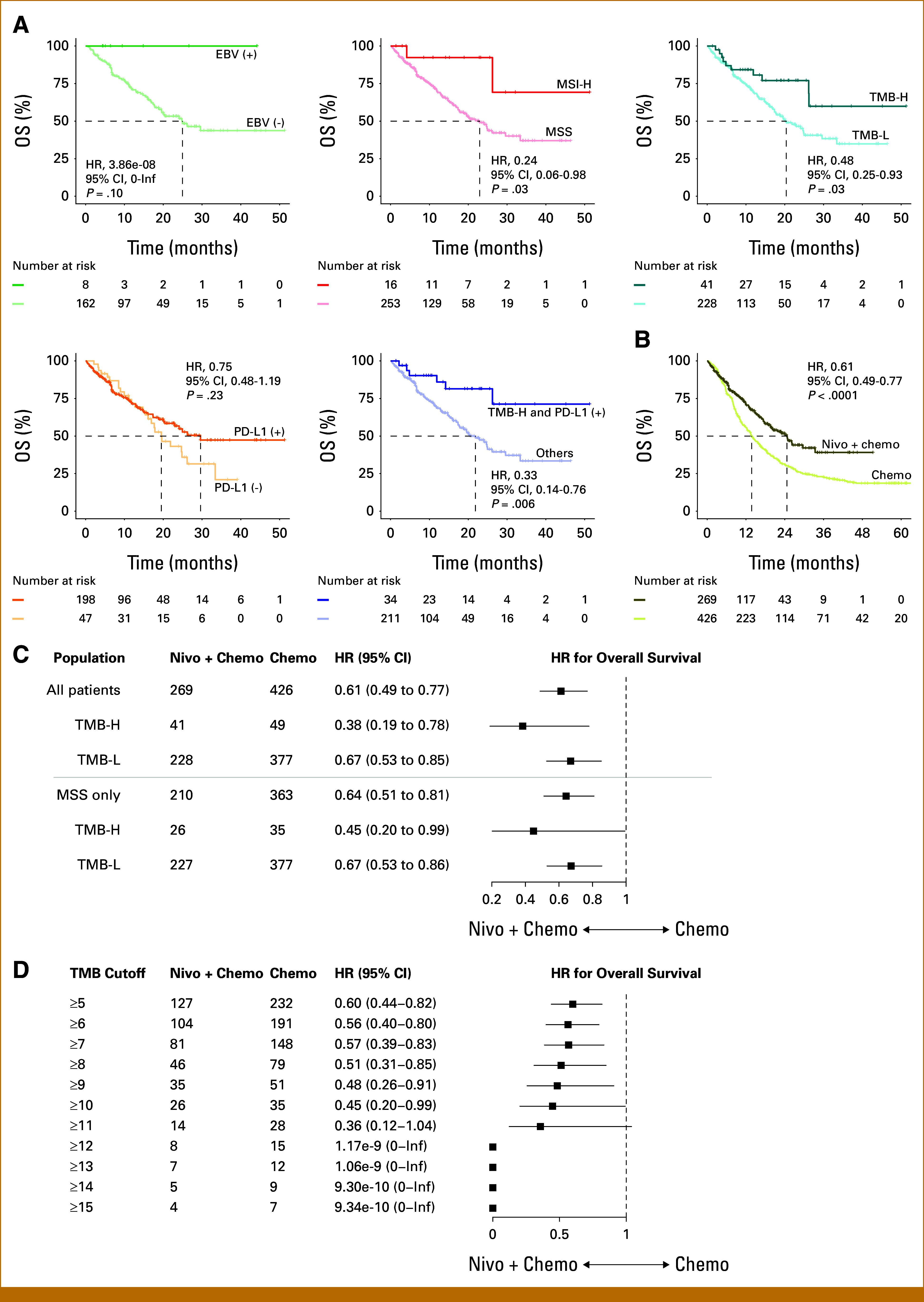
Clinical benefit of first-line nivolumab plus chemotherapy by biomarker status. (A) Kaplan-Meier curves displaying overall survival after first-line nivolumab plus chemotherapy (CAPOX or FOLFOX), stratified by biomarker-defined subgroups. TMB-H was defined as ≥10 mut/Mb, and TMB-L as <10 mut/Mb. HRs with 95% CIs are from Cox proportional-hazards models. *P* values are from two-sided log-rank tests. “Others” denotes single-positive and double-negative cases for the corresponding biomarker pair. (B) Kaplan-Meier curves displaying 5-year overall survival, comparing first-line nivolumab plus chemotherapy (Nivo + chemo) versus chemotherapy alone (CAPOX or FOLFOX; chemo) groups. (C) Forest plot displaying clinical benefit of first-line nivolumab plus chemotherapy versus first-line chemotherapy alone by TMB and MSI status. (D) Forest plot showing clinical benefit of first-line nivolumab plus chemotherapy versus first-line chemotherapy alone across different TMB cutoffs. For TMB cutoffs ≥12 mut/Mb, 95% CIs could not be estimated because of the absence of death events in the nivolumab plus chemotherapy group, resulting in infinite upper bounds. The analysis was restricted to MSS tumors. CAPOX, capecitabine and oxaliplatin; FOLFOX, infusional fluorouracil, leucovorin, and oxaliplatin; HR, hazard ratio; Inf, infinity; MSI, microsatellite instability; MSS, microsatellite stable; TMB, tumor mutational burden; TMB-H, high TMB; TMB-L, low TMB.

Among well-established predictors of ICI outcomes,^31-35^ EBV status was not significantly associated with overall survival in patients treated with nivolumab plus chemotherapy although a favorable trend was observed (hazard ratio [HR], 3.86e-08 [95% CI, 0 to infinity]; log-rank test *P* = .10; Fig 3A). Patients with MSI-H tumors exhibited longer overall survival than those with MSS tumors (HR, 0.24 [95% CI, 0.06 to 0.98]; log-rank test *P* = .03). When stratified by TMB level, using the FDA-approved cutoff of 10 mutations per megabase (mut/Mb),^36^ patients with high TMB (TMB-H) displayed improved overall survival relative to those with low TMB (TMB-L; HR, 0.48 [95% CI, 0.25 to 0.93]; log-rank test *P* = .03). For PD-L1 expression, a trend toward better survival was observed in PD-L1–positive patients although the difference did not reach statistical significance (HR, 0.75 [95% CI, 0.48 to 1.19]; log-rank test *P* = .23). Importantly, when TMB and PD-L1 status were evaluated in combination, patients who were double-positive (TMB-H– and PD-L1-positive) experienced a significantly longer overall survival compared with single-positive or double-negative groups (HR, 0.33 [95% CI, 0.14 to 0.76]; log-rank test *P* = .006). These findings collectively suggest that well-established predictors of ICI could also predict clinical benefit among patients treated with nivolumab plus chemotherapy.

### TMB-Dependent Benefit of Nivolumab Plus Chemotherapy Versus Chemotherapy Alone

Next, we evaluated the real-world therapeutic benefit of first-line nivolumab plus chemotherapy (n = 269) compared with first-line chemotherapy alone (CAPOX or FOLFOX, n = 426) and simultaneously assessed whether this benefit differed according to the TMB level as suggested by recent exploratory biomarker analyses from the CheckMate 649 trial.^13^ We initially evaluated the benefit of nivolumab plus chemotherapy over chemotherapy alone across all patients according to TMB subgroups (TMB-H and TMB-L) and subsequently restricted the analysis to MSS tumors. Given that MSI-H is already an established predictor of ICI response and is highly correlated with elevated TMB,^37^ inclusion of MSI-H cases would confound the assessment of TMB's incremental clinical utility in the broader patient population. This approach aligns with exploratory analyses from CheckMate 649, which similarly examined TMB effects within MSS subgroups to identify patients who may benefit from chemoimmunotherapy beyond those with MSI-H disease.^13^

We confirmed a clear benefit of nivolumab plus chemotherapy over chemotherapy alone (HR, 0.61 [95% CI, 0.49 to 0.77], log-rank test *P* < .0001; Fig 3B). In the multivariable analysis adjusting for potential confounders (Data Supplement, Table S1), the combination of chemotherapy and nivolumab was independently associated with improved overall survival compared with chemotherapy alone (adjusted HR, 0.59 [95% CI, 0.43 to 0.80]; Data Supplement, Fig S3). When stratified by TMB, the survival advantage was more evident in patients with TMB-H tumors (HR, 0.38 [95% CI, 0.19 to 0.78]) than in those with TMB-L tumors (HR, 0.67 [95% CI, 0.53 to 0.85]; Fig 3C). In analyses restricted to MSS tumors, the benefit of nivolumab plus chemotherapy remained significant (HR, 0.64 [95% CI, 0.51 to 0.81]). Notably, favorable outcomes were consistently observed in both MSS/TMB-H and MSS/TMB-L subgroups (HRs, 0.45 and 0.67 [95% CIs, 0.20 to 0.99 and 0.53 to 0.86, respectively]), in line with trends reported in CheckMate 649,^13^ while demonstrating a statistically significant benefit in the MSS/TMB-H subgroup. Genomic profiling of the MSS/TMB-H population revealed a mixed TCGA-subtype architecture bridging CIN, EBV, and GS hallmarks,^6^ with baseline alterations being statistically balanced between the two treatment groups (Data Supplement, Fig S4).

Given that the current FDA approval of ICIs based on an absolute TMB cutoff of ≥10 mut/Mb may be overly broad,^38^ we further investigated the relationship between TMB and the therapeutic benefit of nivolumab plus chemotherapy versus chemotherapy alone among patients with MSS tumors. To refine patient stratification, we sequentially increased the TMB cutoff in 1-mut/Mb increments starting from 5 mut/Mb and evaluated the overall survival benefit of nivolumab plus chemotherapy versus chemotherapy alone at each cutoff among patients with MSS tumors. Although statistical significance was not consistently achieved, a monotonic association was observed between TMB cutoffs and improved survival benefit from nivolumab plus chemotherapy (Fig 3D). The trend became evident at a cutoff of 12, where the overall survival advantage of nivolumab plus chemotherapy was clearly maintained, with no deaths observed in the nivolumab plus chemotherapy group at TMB ≥ 12. These findings suggest that patients with higher TMB derive greater clinical benefit from nivolumab plus chemotherapy.

### Gene-Specific Associations With Overall Survival by Treatment Context

**FIG 4.**
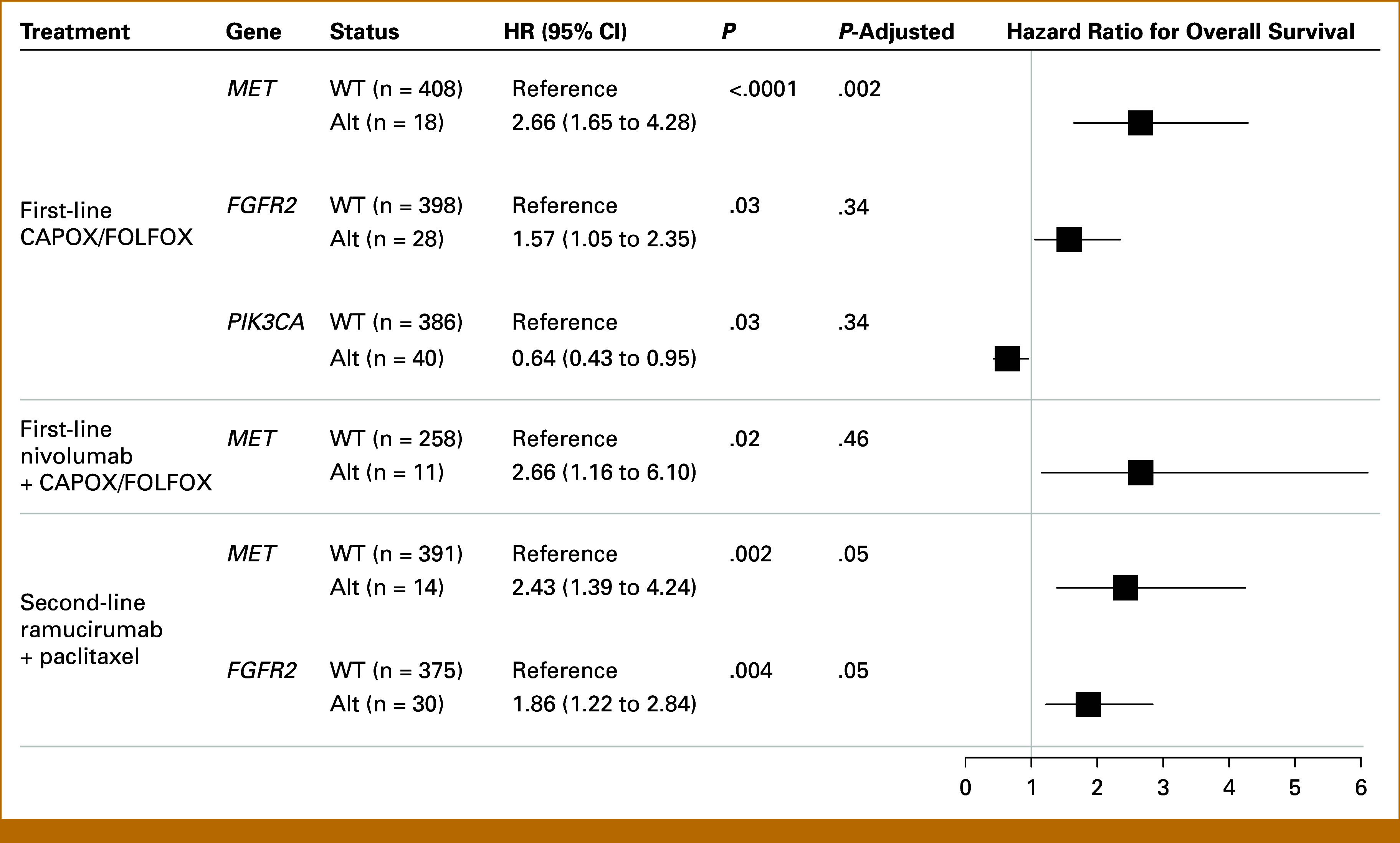
Gene-specific associations with overall survival by treatment context. Forest plot comparing overall survival of patients stratified by Alt versus WT status across recurrently Alt genes in different treatment settings. Only comparisons with statistically significant *P* values derived from Cox regression analysis are displayed. Alt, altered; WT, wild-type.

To explore the prognostic relevance of oncogenic alterations in different therapeutic contexts, we evaluated the association between recurrently altered genes (≥10 patients harboring oncogenic alterations in each treatment group) and overall survival across the three most common treatment regimens in our cohort (Fig 1A): (1) first-line CAPOX or FOLFOX, (2) first-line nivolumab plus CAPOX or FOLFOX, and (3) second-line ramucirumab plus paclitaxel. These analyses were performed in an exploratory framework, with associations interpreted primarily based on effect sizes and corresponding 95% CIs. In the first-line CAPOX or FOLFOX group (n = 426), *MET* and *FGFR2* alterations were associated with unfavorable survival outcomes (HRs, 2.66 and 1.57 [95% CIs, 1.65 to 4.28 and 1.05 to 2.35, respectively]; Fig 4), whereas *PIK3CA* mutations were linked to longer survival (HR, 0.64 [95% CI, 0.43 to 0.95]). Among patients treated with first-line nivolumab plus chemotherapy (n = 269), *MET* alterations were associated with shortened overall survival (HR, 2.66 [95% CI, 1.16 to 6.10]). In the second-line ramucirumab plus paclitaxel regimen (n = 405), *MET* and *FGFR2* alterations were unfavorable prognostic markers (HRs, 2.43 and 1.86 [95% CIs, 1.39 to 4.24 and 1.22 to 2.84, respectively]), as in the first-line CAPOX or FOLFOX group.

## DISCUSSION

The therapeutic landscape of advanced gastric cancer has been reshaped by recent regulatory approvals of biomarker-driven therapies, including ICIs for PD-L1–positive and MSI-H tumors, trastuzumab and trastuzumab deruxtecan for HER2-positive tumors, and zolbetuximab for CLDN18.2-expressing tumors.^4^ However, maximizing the clinical benefit of these approaches requires deeper insight into the interplay between genomic alterations and established biomarkers. RWD from routine clinical practice may be critical to this effort, capturing the heterogeneity of patient populations, treatment patterns, and outcomes that extend beyond the controlled environments of prospective clinical trials. In this context, we present one of the largest real-world genomic analyses of metastatic gastric cancer in the East Asian population, integrating clinical sequencing data from 1,283 Korean patients with comprehensive biomarker profiling and treatment outcomes.

This study identified multiple genes associated with established biomarkers, including HER2, EBV, PD-L1, and CLDN18.2 status. These associations—including the co-occurrence of *CCNE1* and *MYC* with HER2 amplification, as well as the enrichment of *PIK3CA* in EBV-positive and *MET* in CLDN18.2-positive tumors—highlight the distinct molecular features of biomarker-defined subgroups. Furthermore, several genomic alterations demonstrated treatment-context-dependent prognostic effects: *PIK3CA* alterations were associated with favorable outcomes in the first-line chemotherapy setting, whereas *MET* and *FGFR2* alterations were linked with poor survival across multiple treatment contexts. Collectively, these findings provide a rationale for the development of combination therapies and refined patient stratification strategies in future clinical trials.

Our analysis also revealed a significant enrichment of *CDH1* alterations in young female patients (younger than 45 years). Given the technical limitations of tumor-only sequencing in fully distinguishing somatic from germline variants, this enrichment likely includes residual *CDH1* germline variants after the variant filtering procedure. Indeed, the demographic pattern we observed strongly aligned with the known epidemiology of hereditary diffuse gastric cancer (HDGC), where *CDH1* germline mutations are well-established as the major genetic cause with an average age of 38 years for clinical presentation.^39^ Interestingly, a study showed that early-onset lobular breast cancer may be the first manifestation of HDGC in female carriers, suggesting the sex-dependent cancer risks associated with *CDH1* germline mutations.^40^ A recent large-scale genomic study in East Asian populations has reported that *CDH1* germline variants are present at higher-than-expected prevalence (7.4%) in Japanese patients with gastric cancer.^41^ The shared distribution of recurrent *CDH1* variants between Japanese and Korean populations suggests common ancestral components among East Asians.^41,42^ Furthermore, several studies have documented *CDH1* germline mutations in both familial and sporadic early-onset gastric cancer cases in Korea, highlighting the clinical relevance of *CDH1* screening in high-incidence populations.^43,44^

Consistent with a previous study,^13^ we confirmed a clear clinical benefit of first-line nivolumab plus chemotherapy in patients with TMB-H tumors. Although the FDA approval of pembrolizumab for TMB-H solid tumors uses a cutoff of ≥10 mut/Mb based on the KEYNOTE-158 trial,^36^ our data suggest that a higher threshold may be more appropriate for identifying patients who derive substantial benefit from chemoimmunotherapy. This discordance raises three important concerns regarding TMB assessment and interpretation. First, ancestry-driven recalibration of TMB may be required to account for the population structure. Recent analyses have shown that TMB estimates from tumor-only sequencing substantially overclassify patients of Asian and African ancestries into the TMB-H category because of germline variant contamination.^45^ Therefore, TMB-H status was significantly associated with improved outcomes only in patients of European ancestry, underscoring the need for ancestry-specific recalibration of TMB in non-European populations.^45^ Second, technical variability in TMB estimation across targeted sequencing panels and bioinformatics pipelines could introduce substantial imprecision. The Friends of Cancer Research TMB Harmonization Project has revealed that differences in panel size, gene content, and bioinformatic pipelines are major drivers of variability in TMB estimates.^46^ While targeted sequencing panels provide cost-effective alternatives to whole-exome sequencing (WES), the narrow sequencing region necessitates careful extrapolation of global mutational burden, and correlation with WES-derived TMB varies widely across panels and cancer types.^47,48^ Third, the FDA-approved threshold of ≥10 mut/Mb may be overly broad^38^ and require further refinement. The KEYNOTE-158 data underlying FDA approval showed that response rates varied markedly across TMB thresholds: 6.7% for <10 mut/Mb, 12.5% for 10-13 mut/Mb, and 37% for >13 mut/Mb, suggesting that the 10 mut/Mb cutoff does not effectively separate responders from nonresponders.^49^ Notably, median overall survival was actually longer in the TMB < 10 cohort (13 months [95% CI, 11.5 to 14.6]) compared with the TMB ≥ 10 cohort (11.7 months [95% CI, 8.2 to 19.1]), raising questions about the clinical utility of this cutoff.^49^ Altogether, while the current TMB ≥ 10 mut/Mb cutoff remains capable of stratifying patients receiving chemoimmunotherapy into a benefit group, our finding that benefit becomes evident at TMB ≥ 12 mut/Mb cutoff supports emerging evidence that context-specific redefinition of TMB thresholds may be warranted.

This study has several limitations: (1) the use of two different targeted sequencing panels could introduce potential bias stemming from differences in gene content^50^; (2) by design, targeted sequencing captures a highly restricted genomic region, limiting the assessment of broad-scale genomic events such as tumor aneuploidy, consequently precluding formal classification of TCGA-defined molecular subtypes such as CIN and GS; and (3) as illustrated by the *CDH1* case, current reference resources implemented in germline variant filtration may insufficiently represent East Asian populations, increasing the risk that germline variants are not fully discarded in tumor-only sequencing workflows. These constraints underscore the need for larger East Asian reference data sets, systematic cross-panel harmonization, and further analyses to validate and extend our findings.

In this large, real-world Korean gastric cancer cohort, we delineated the genomic landscape across biomarker-defined subgroups and demographics, confirming recurrent altered genes and the enrichment of *CDH1* in younger female patients. High mutational burden was associated with greater benefit from first-line nivolumab plus chemotherapy, and several genomic alterations showed prognostic effects across treatment contexts. Together, these findings support risk-adapted screening and biomarker-guided therapeutic selection to advance precision oncology in gastric cancer.

## Data Availability

The data generated in this study are not publicly available owing to institutional data-sharing restrictions.
